# Oligometastatic Cancer: Key Concepts and Research Opportunities for 2021 and Beyond

**DOI:** 10.3390/cancers13112518

**Published:** 2021-05-21

**Authors:** Petr Szturz, Jan B. Vermorken

**Affiliations:** 1 Medical Oncology, Department of Oncology, Lausanne University Hospital (CHUV), 1011 Lausanne, Switzerland; szturz@gmail.com; 2 Department of Medical Oncology, Antwerp University Hospital, 2650 Edegem, Belgium; 3 Faculty of Medicine and Health Sciences, University of Antwerp, 2610 Antwerp, Belgium

Traditionally, clinicians distinguished three forms of cancer outgrowth. Classification in early, locally or locoregionally advanced, and metastatic disease had purposefully reflected patient prognosis and treatment options. More recently, we learned that some molecular features, such as positivity for human papillomavirus (HPV) in oropharyngeal cancer, may partially equalize prognostic differences between these categories, particularly between early and locoregionally advanced stages [1]. There are more examples demonstrating imperfections of simplified models used in clinical practice if not supported by a solid understanding of disease biology. It is therefore intriguing to speculate on molecular mechanisms that can cut out a piece of the metastatic spectrum and set a specific situation in oncology known as oligometastatic cancer. Usually defined by the presence of one to five distant lesions safely treatable with local approaches, it can be considered an intermediate state between a locoregional and typical polymetastatic disease (Figure 1) [2,3]. Mounting evidence has demonstrated that this distinction is based on biological characteristics including genetic determinants (e.g., PBRM1 mutations), epigenetic modifiers (e.g., overexpression of 14q32-encoded miRNAs), and immune response markers (such as CD3^+^ and CD8^+^ T-cell infiltration) [4].

The clinical concept of the oligometastatic state has its origins in metastasectomies performed in the 1920s and 1930s. In the following 50 years, complete resection of hepatic metastases from colorectal carcinomas and pulmonary metastases from sarcomas and renal cell cancers emerged as a potentially curative intervention. In the 1990s, stereotactic (ablative) body radiotherapy (SABR/SBRT) developed as an alternative to metastasectomy, and around the same time, Hellman and Weichselbaum coined the term “oligometastases”. Parallelly, radiofrequency ablation was modified to be applied percutaneously and used to treat liver tumours under radiological guidance. Further methods were also made available in oncology, including cryotherapy, lasers, microwave hyperthermia, high-intensity focused ultrasound, and ethanol injections [5,6]. Subsequently, moving from the period of nosological evolution and therapeutic development, we have entered a new era aiming at reliable prediction and individualization.

Oligometastatic disease has been in the spotlight of researchers, with a markedly growing number of new scientific papers every year (Figure 2) and even penetrating into the latest staging system of the American Joint Committee on Cancer (AJCC) for non-small cell lung cancer [1]. This is not surprising because it has been shown that local ablation of limited metastases can lead to prolonged disease control and eventually cure, such as in some oligometastatic HPV-positive oropharyngeal cancer cases. However, there are more objectives to attain and more indications and scenarios to intervene. Not yet standardized but already adopted by numerous investigators, the current terminology restricts the use of “oligometastasis” to newly diagnosed cases (synchronously with the primary tumour), while the term “oligorecurrence” should be reserved for a metachronous dissemination (> 3-6 months after the primary cancer diagnosis) or to a new manifestation in patients with a history of a prior metastatic disease. In those undergoing anticancer treatment, we may speak of “oligoprogression” if few distant lesions grow or “oligopersistence” if disease control has been achieved (Figure 1) [2,3]. Therefore, local ablation has an important position both as an adjunct to and a replacement of systemic treatment in the palliative setting, which is reinforced by its generally advantageous toxicity profile.

Compared with a leukaemia-like dissemination of many malignant processes, the encouraging aspect of a spatially limited oligometastatic disease has ignited a steep rise of scientific interest with new research questions and challenges. We have divided them in three main categories, summarized in Table 1 [2,3,4,7,8,9,10,11,12,13,14,15,16,17,18,19,20,21,22,23,24,25,26,27,28,29,30]. One of the major drawbacks of the oligometastatic concept is the current perception relying on therapeutic opportunity and cross-sectional imaging rather than intra- and intercellular processes [7]. This can be partially bypassed by refining diagnostic criteria in that we include disease kinetics measures, molecular imaging, and predictive nomograms, but sooner or later a better understanding of disease biology will become indispensable for further progress [4,7,10,11,12,13]. If we accept the contemporary definition and its shortcomings, the next step is to determine the optimal treatment strategy. The body of existing evidence, especially from phase III trials, is far from satisfactory, and improvements are already needed at the level of study design with respect to clinical endpoints and the choice of modalities [9,24,29]. The latter aspect may indeed be crucial not only due to the large range of possible treatment options and indications, but also because of various combinations and sequencing schedules [9,14,15,16,17,18]. It is therefore noteworthy that local ablation should not be used indiscriminately in the oligometastatic setting. The decision making is a multistep process influenced by the technical feasibility of a given procedure and clinical judgement of its relevance based on patient-related and disease-related factors [5]. Moreover, some of the positive randomized trials, including the CLOCC trial investigating radiofrequency ablation and the SABR-COMET trial evaluating stereotactic radiotherapy, were criticised for imbalances between the treatment arms. Further doubts have been raised following the publication of the PulMiCC trial which did not find a benefit of pulmonary metastasectomy over active monitoring in 65 colorectal cancer patients [30].

Despite these controversies and information gaps, local approaches should be praised for their cost-effectiveness, compliance, and low toxicity [21,24,30]. Moreover, oligometastases provide a new and exciting area of scientific opportunities. On the other hand, it is difficult to anticipate their long-term development, as non-invasive modalities may ultimately be preferred, which is also being fuelled by the enormous amount of research invested in modern systemic treatments and accompanying predictive biomarkers. However, technical advances increasing the availability and accessibility of local interventions may revolutionize their use in smaller practices. Already now they are prioritized in certain clinical situations and represent a viable alternative for some patients according to their individual preferences. Local therapies may eventually become inseparable parts of selected anticancer protocols, but will for sure retain their relevance in cases refractory to systemic therapies and in resource-limited countries remaining out of reach of the latest medicines.

## Figures and Tables

**Figure 1:**
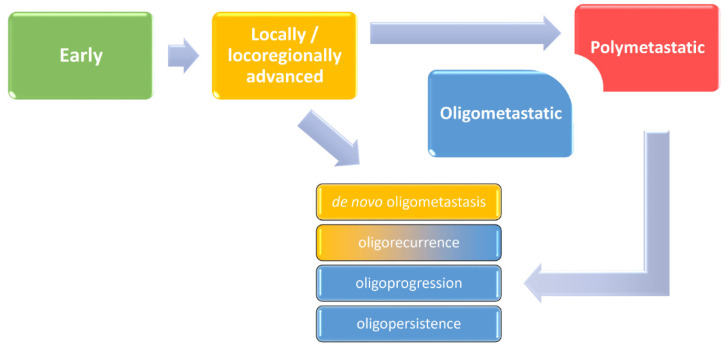
Development of clinically overt malignant disease with a possibility of oligometastatic cancer, as an intermediate state between locoregionally advanced and typical metastatic disease, and its different forms.

**Figure 2:**
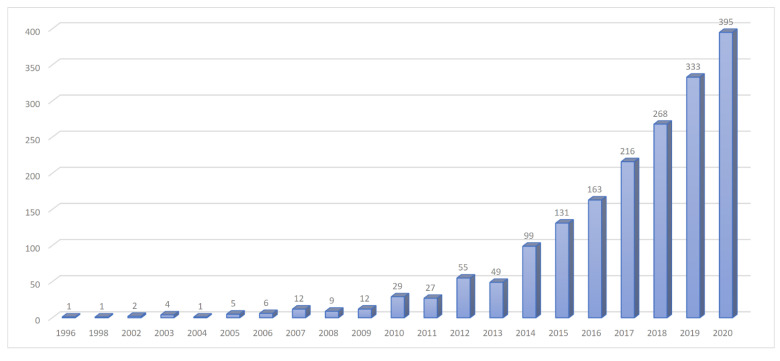
Increasing number of scientific papers on oligometastatic disease per year from 1996 to 2020. Results correspond to the search term “oligometastatic” in the National Library of Medicine on 4 April 2021 (https://pubmed.ncbi.nlm.nih.gov).

**Table 1:** An overview of key concepts and research opportunities in oligometastatic cancer.

Domain	Issue	Challenges	Progress and Objectives
Biology	Determinants of oligometastatic phenotype	Genetic, epigenetic, and immune factors	PBRM1 mutations, overexpression of 14q32-encoded miRNAs, CD3+ and CD8+ T-cell infiltration [4]
		Definition based on disease biology rather than therapeutic opportunity	Driver mutations, miRNAs, somatic copy number alterations, intratumour heterogeneity [7]
	Modifiers of oligometastatic phenotype	Microenvironment	Granzyme-B+ T-cell infiltration [8]
		Abscopal effect as an in vivo vaccine	Concurrent use of SABR and immunologic agents [9]
Workup	Clinical determinants of oligometastatic phenotype	Tumour burden	Number (≤3–5), size (≤5 cm), and localization of lesions (≤3–5 organs) [2,10]
		Disease kinetics	Clinical factors (DFS) and biological factors (miRNAs, intratumour heterogeneity, somatic copy-number alterations) [10]
		Disease subtypes	Synchronous / metachronous, repeat / induced, oligometastasis, oligorecurrence, oligoprogression, oligopersistence [3]
		Prognostic factors	Nomograms (e.g., the Metabank score for SABR), DFS, tumour markers, baseline interleukin 1α [7,11,12]
	Molecular imaging	New PET tracers	PSMA, Her-2, ^68^Ga-FAPI, PD-L1 [10,13]
	Liquid biopsy	Posttreatment follow-up	ctDNA burden, oligoclonal expansion [12]
Treatment	Optimization	Combinations with systemic therapy	Immune checkpoint inhibitors [9,14]
		Consolidation with local ablation	Oligometastatic disease after first-line systemic treatment (induced oligopersistence) [15]
		Radical treatment of the primary tumour in the setting of synchronous oligometastases	Radiotherapy in nasopharyngeal cancer, surgery in non-small cell lung cancer [16,17]
		Treatment sequencing	Systemic treatment before or after local ablation [18]
	Innovation	Reversal of metastatic to oligometastatic phenotype	Epigenetic modifiers [10]
		New technologies	Magnetic resonance-guided radiotherapy, single-cell sequencing, Holmium-166 microsphere selective radioembolization [9,19,20]
	Adaptation (e.g., pandemic scenario)	Rationalization of fractionation schedules	Ultra-high single-dose radiotherapy (24 Gy) [21]
		Bridging therapy	Radical radiotherapy to delay curative surgery [22]
	Individualization	Predictive factors	Baseline immune phenotype and tumour mutation status [13]
		Biological age	SABR in unfit elderly patients [23]
		Choice of a local ablation tool	Patient-, disease-, resource-, and experience-related factors [9,24]
	Expansion	New indications	e.g., pancreatic cancer [25]
		Paediatric patients	Sarcomas (e.g., rhabdomyosarcoma) [26]
		Upfront local ablation in polymetastatic disease	Cytoreduction, elimination of immunotherapy- and TKI-resistant clones, enhancement of tumour antigen presentation and immunogenicity [18]
	De-escalation	In combination with immunotherapy	Worse local control with low doses of SABR (<60 Gy) [14]
	Timing	Synchronous vs. metachronous oligometastases	Better outcomes in the metachronous setting [11]
	Benefits and drawbacks	Rare adverse events	Thermal ablation and cryoablation of liver metastases, embolization [24]
		Patient reported outcomes	Pain response and quality of life [27]
		Cost-effectiveness	SABR more cost-effective than systemic therapy [28]
		Resource-limited countries	SABR, particularly single-dose radiotherapy [21,28]
	Clinical trial design	New endpoints adjusted to local ablation	Corrected DFS, Time to New Systemic Therapy, WideSpread Progression-Free Survival [29]
		Addressing ambiguous results	Local ablation of pulmonary metastases in colorectal cancer [30]

**Abbreviations:** PET, positron emission tomography; PBRM1, polybromo 1; miRNA, micro ribonucleic acid; CD, cluster of differentiation; SABR, stereotactic ablative body radiotherapy; DFS, disease-free survival; PSMA, prostate-specific membrane antigen; Her, human epidermal growth factor receptor; ^68^Ga-FAPI, gallium-68 fibroblast activation protein inhibitor; PD-L1, programmed death-ligand 1; ctDNA, circulating tumour deoxyribonucleic acid; TKI, tyrosine kinase inhibitor.
